# Validation of simulator-based neuroangiographical training

**DOI:** 10.1016/j.bas.2026.105931

**Published:** 2026-01-08

**Authors:** Elle Vermeulen, Ramon Torné, Ebba Katsler, Nuri Alioski, Mihail Petrov, Teodora Sakelarova, Leire Pedrosa, Torstein Ragnar Meling, Nikolay Velinov, Hieronymus Damianus Boogaarts

**Affiliations:** a Department of Neurosurgery, University Hospital of Brussels, Laarbeeklaan 101, 1090, Jette, Belgium; b Department of Neurosurgery, Hospital Clinic of Barcelona, C/ Villarroel, 170, 08036, Barcelona, Spain; c Department of Neurosurgery, National Hospital of Denmark (Rigshospitalet), Blegdamsvej 9, 2100, Copenhagen, Denmark; d Department of Neurosurgery, “Saint Anna” Hospital, Sofia, Bulgaria; e Department of Neurosurgery, University Multiprofile Hospital for Active Treatment with Emergency Medicine “N. I. Pirogov”, Sofia, Bulgaria; f Instituto de Investigaciones Biomédicas August Pi i Sunyer (IDIBAPS), Barcelona, Spain; g Department of Neurosurgery. Radboud University Medical Center, Geert Grooteplein Zuid 10, 6500, HB, Nijmegen, the Netherlands

**Keywords:** Simulator, Neurosurgical training, Angiography

## Abstract

**Background:**

Angiography is a diagnostic and interventional technique in (endo)vascular neurosurgery that demands a high level of precision and technical expertise. Traditionally, mastery of angiographic techniques has relied on hands-on training, often limited by patient availability and procedural complexity. This study aims to investigate the effectiveness of simulator-based training for angiography, determining its role in enhancing procedural proficiency and its potential integration into a neuroendovascular training curriculum.

**Research question:**

Is a simulator-based training for neuroangiography effective ?

**Materials and methods:**

Participants (22 trainees and 10 experts) were recruited in neurosurgical departments from four international university hospitals. After a familiarization session, each participant performed 6 attempts of diagnostic angiography and 3 attempts of coiling on an Angio mentor endovascular simulator. Data gathered were procedure time, fluoroscopy time, amount of contrast injected, number of roadmap sequences and number of errors. The learning curve was studied and contrasting group assessment was performed.

**Results:**

There was a clear steep improvement for all parameters in the learning curve which flattens out as the trainees master angiography and coiling. Trainees had a notable reduction in procedure time, approaching the experts' levels after sixth and seventh attempt of diagnostic angiography. The contrasting group assessment demonstrated discriminating results of experts compared to trainees and a distinctly increasing overlap between trainees and experts with increasing number of attempts.

**Discussion and conclusions:**

Endovascular simulators enable skill acquisition in a controlled environment, enhancing technical proficiency in neuroendovascular coiling and angiography, and should play a role in neuroendovascular training.

## Highlights

• There is a steep learning curve for the trainees for the performance of a diagnostic angiography and coiling on endovascular simulators
• Endovascular simulators are valid tools to demonstrate expertise in neuroangiography
• Endovascular simulators enable skill acquisition in a controlled environment
• Endovascular simulators should play a role in neuroendovascular training.

## 1 Introduction

Endovascular techniques play an increasingly important role in treatment of patients with cerebrovascular pathology. Hence, leading to a higher demand for physicians trained in neuroendovascular techniques. Cerebral angiography, a cornerstone diagnostic and interventional technique, demands a high level of precision, technical expertise, and procedural understanding to ensure patient safety and optimal outcomes. Traditionally, the mastery of angiographic techniques has relied heavily on gradual hands-on training during live cases in the hybrid operating room or endovascular suite, often limited by patient availability, procedural complexity, and ethical considerations (Li et al., 2023). With the increasing complexity of angiographic interventions and the demand for well-trained operators, there is a growing need for innovative and effective training methods.

Simulator-based training has emerged as a transformative tool in medical education, offering a risk-free environment for learners to acquire and refine technical skills (Zaika et al., 2023; Patchana et al., 2020). While simulators are widely utilized by various surgical specialists, they are not yet universally incorporated into the neuroendovascular field (Walsh et al., 2015; Kreiser et al., 2021). By replicating real-life procedural scenarios, simulators enable trainees to practice vascular access, catheter manipulation, and contrast injection techniques with iterative feedback and without the immediate pressures of a clinical setting. Furthermore, this modality allows standardization of training, the development of procedural competency, and the assessment of performance in a controlled and reproducible manner (Pannell et al., 2016; Yi et al., 2023).

This study aims to investigate the effectiveness of simulator-based training for angiography, focusing on its impact on technical skill acquisition and knowledge retention using the ANGIO Mentor by Surgical Science simulator among neurosurgical residents with minimal exposure to endovascular procedures. Results of training metrics are compared to experienced interventionalists. By evaluating the outcomes of simulation-based learning, this research seeks to determine its role in enhancing procedural proficiency and its potential integration into the neuroendovascular training curriculum.

## 2 Methods

This prospective multicenter study consists of an endovascular training cycle conducted over one year in four European centers: University Multiprofile Hospital for Active Treatment with Emergency Medicine “N. I. Pirogov”, Sofia (Bulgaria); Hospital Clinic of Barcelona, Barcelona (Spain); Radboud medical center, Nijmegen (Netherlands) and the National Hospital of Denmark, Copenhagen (Denmark).

We included at minimum 5 neurosurgical residents with no prior training in endovascular therapy (trainees) in each center and 10 experienced neuro-interventionalists (experts) across all centers. Experts were defined as physicians with more than 2 years of experience in neuroendovascular treatments. A participant specific ID was assigned to each participant once included into the study. The results were anonymized by recording the data to the participant ID.

A local expert study coordinator supervised all simulation sessions in their respective center. There was specific training for each local coordinator so that they supervised participants in a maximally standardized manner. The training was recorded and was digitally available to each local coordinator.

The Angio Mentor simulator was provided by Surgical Science for training in all centers. The Angio Mentor VR training simulator platform provide a comprehensive, safe environment for multi-level hands-on practice of endovascular procedures and techniques performed in the laboratory, interventional suite or operating room.

A well-structured approach was designed to evaluate participants' independent performance and learning outcomes. Participation in the study involved a one-week training program for neuroendovascular procedures in 3 sessions. The first session consisted of self-learning using uniform videos on the use of the Angio Mentor simulator and basic theoretical knowledge for neurointervention. The second session was a hands-on training on the simulator for performing diagnostic angiography of the right internal carotid artery. The third session was hands-on coiling a left carotid T aneurysm. Each hands-on session consisted of one familiarization session, where the participants completed one attempt under the guidance of the local coordinator, who explained in detail the different steps of the exercise. This familiarization session was not recorded for analysis and served solely to accustom participants to the system and procedures.

The following attempts (6 for the diagnostic case and 3 for the coiling case) were analyzed. The attempts were divided in semi-autonomic and autonomic attempts, with gradually less help by the local coordinator (see Table 1). Errors were predefined and registered as minor (oral help) and major (physical help) interventions from the local coordinator (see Table 1). The different steps for each exercise and the materials to be used were standardized for all participants. Detailed overview of the sessions is given in Table 2.

**Table 1:** Overview of one week training program per participant.

**One week training program**				1 WEEK
**Session 1**	**Self-learning with standardized videos**			
**Session 2**	**Diagnostic case (hands-on) -** Right internal carotid artery angiography		DAY 1	
	1 familiarization session	Guidance oral + physical aid (not analyzed)		
	3 semi- autonomic	Pro active oral help + - major interventions^a^		
	3 autonomic	Minor and major interventions^a^		
**Session 3**	**Coiling case (hands-on) -** Left Carotid T aneurysm coiling		DAY 2	
	1 familiarization session	Guidance oral + physical aid (not analyzed)		
	2 semi- autonomic	Pro active oral help + - major interventions^a^		
	1 autonomic	Minor and major interventions^a^		

a Interventions: Major interventions: Physical aid/Minor interventions: The local coordinator needs to advice the participant to advance the guide wire before the catheter, the trainee loses control of the tip (not seen on the screen) and the expert advises needs to adjust the window/fluoroscopy, the trainee loses control of the microcatheter during coiling (e.g. advances in the aneurysm), and the instructor needs to instruct how to stabilize it.

**Table 2:** Overview of the hands-on sessions 2 and 3.

Session 2: Diagnostic case (hands-on) - Right internal carotid artery angiography	
**Goal**	AP and lateral run of right ICA
Catheter	4Fr diagnostic vertebral catheter
Wire	Angled 0.035 inch standard guide wire
Steps	- Wire and catheter in aortic arch - Pull back wire - Roadmap in aortic arch - Wire in brachiocephalic trunk - Catheter in brachiocephalic trunk - Pull back wire - Activate lateral Xray - Roadmap in brachiocephalic trunk - Wire in ICA - Catheter in ICA (pars petrosa) - Angle the c-arm correct (with the skullbase at the middle of the orbit) - Run angiography - Remove catheters during fluoroscopy - Press finish

Session 3: Coiling case (hands-on) - Left Carotid T aneurysm coiling	
**Goal**	3 coils in aneurysm
Catheter	6Fr Multipurpose guide catheter (for diagnostic and treatment)
Wire	Angled 0.035 inch standard guide wire
Microcatheter	SL-10 microcatheter 45° angle
Microwire	Microwire single 2 standard 0.014 inch 45° angle
Coils	3D 360 standard target 12 x 30 = basket 3D 360 soft 8 x 30 = filling 3D 360 soft 7 x 30 = filling
Steps	- Wire and catheter in aortic arch - Pull back wire - Roadmap in aortic arch - Wire in left CCA - Catheter in left CCA - Pull back wire - Activate lateral Xray - Roadmap in left CCA - Wire in left ICA - Catheter in ICA (beginning of pars petrosa) - Pull back wire - Angle the c-arm correct (with the skull base at the middle of the orbit) - Run angiography of left in ICA - Shift to microcatheter and microwire - Zoom in - New roadmap in detail to see aneurysm - Microwire in aneurysm (⅓-⅔) - Microcatheter in aneurysm - Start coiling - after coiling: new angiography to control result - remove catheter out of aneurysm and all equipment during fluoroscopy - Press finish

The training program was scheduled within one week for each participant. The self-learning was sent out at a maximum of a week before. There was at least one day between the angiography and coiling session. All attempts of each session were performed the same day, with 10-min breaks provided approximately every 45 min.

The automatically generated data from the simulator during the hands-on sessions included procedure time, fluoroscopy time, amount of contrast injected, number of roadmap sequences, and catheter advancement without a guide wire. Interventions were manually registered by the local study coordinator and included. Examples of minor interventions were: oral instruction to the trainee to advance the guidewire before the catheter, loss of control of the tip (not seen on the screen), loss of control of the microcatheter during coiling (e.g. advances in the aneurysm).

Statistical analyses were performed using IBM SPSS Statistics for Windows, Version 29.0 (IBM Corp., Armonk, NY). Continuous variables were expressed as mean ± standard deviation (SD) or median with interquartile range (IQR) based on data distribution. Categorical variables were presented as frequencies and percentages. Comparisons between experts and trainees were conducted using Student's t-test for means of continuous variables, and chi-square test for categorical variables. A p-value of <0.05 was considered statistically significant. The first familiarization session was left out in our analyses. Since the study was exploratory no power calculation was performed. Assessments of contrasting groups were performed to differentiate the trainees from the experts (Jørgensen et al., 2018).

## 3 Results

We included 22 trainees and 10 experts across different European centers. Among the trainees, there was a fairly even distribution of men and women (9 and 13, respectively). The majority of the trainees (64 %) were between the ages of 25 and 30. Nineteen trainees had no previous experience with neurointervention, and 3 participants had attended a maximum of 2 courses. The experts were predominantly male (90 %) and all above 30 years of age. More detailed characteristics of the participants are presented in Table 3.

**Table 3:** characteristics of participants.

		Trainee	Expert
Total		22	10
Age	25–30	14	0
	30–40	7	2
	>40	1	8
Gender	Male	9	9
	Female	13	1
Experience with neurointervention	No experience	19	0
	Experience in maximum 2 courses	3	0
	Practicing arteriography and interventions (minimum 2 years)	0	10
Experience with medical simulator	No	16	2
	Yes	6	8

**Fig. 1:**
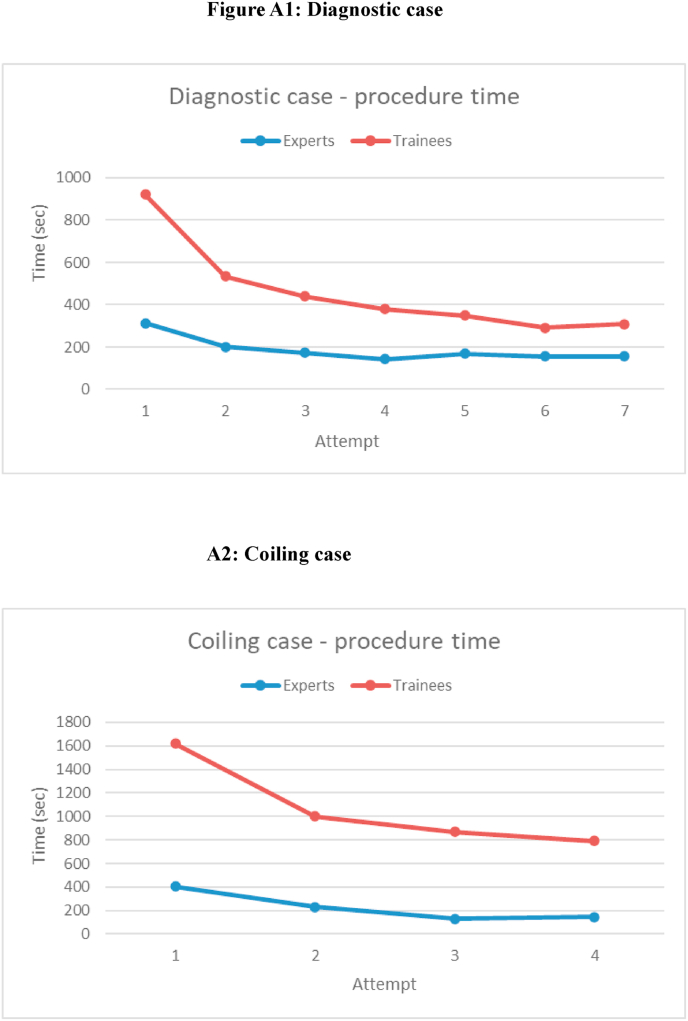
Procedure time.

**Fig. 2:**
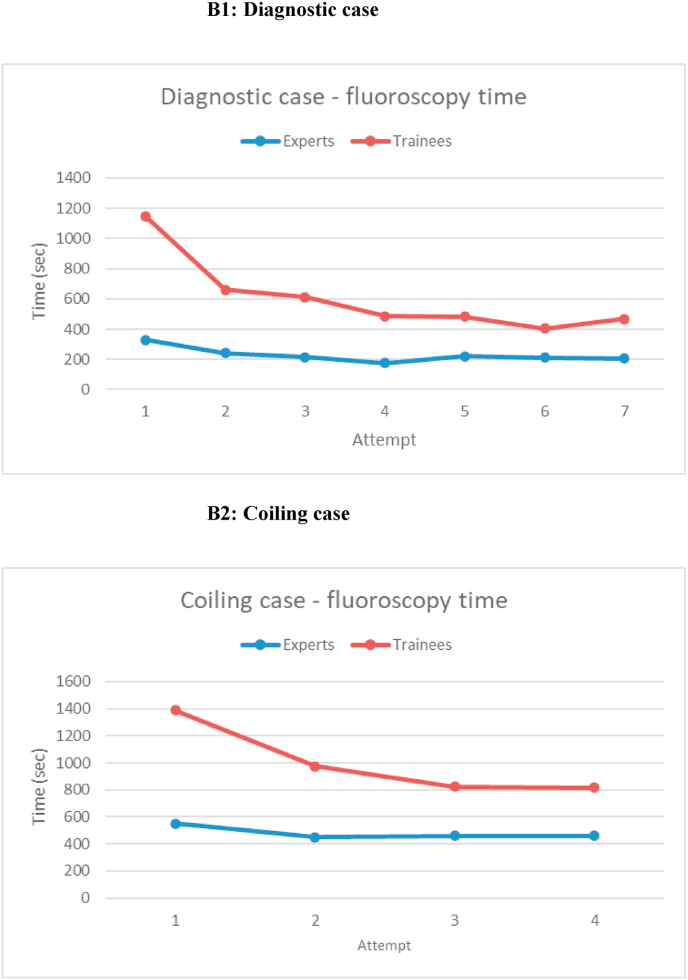
Fluoroscopy time.

The procedure and fluoroscopy time for the trainees' initial attempts was significantly longer compared to the expert's (p < 0.001). However, with trainee's subsequent attempts, there was a notable reduction in procedure time, approaching the levels of the experts after the sixth attempt of diagnostic angiography (but still statistically significantly different, p < 0.001) (see Fig. 1, Fig. 2). This marked improvement has been observed for both the coiling and angiography cases. However, statistically significant differences in the performances between experts and trainees were evident for all attempts (see Fig. 1, Fig. 2).

For both the angiography and coiling case there were no clear learning curves for contrast use and unsafe travel (catheter before the guidewire), no statistically significant differences were found for these parameters between experts and trainees. The number of roadmaps was the same in both groups on both exercises, as this was standardized in the protocol.

In the expert results, all variables remained consistent across each attempt, showing no statistically significant decrease in either the procedure- or fluoroscopy time (see Fig. 1, Fig. 2).

The errors, interventions by the local study coordinators were variable among participants and centers. On average, the trainees had 2 times more interventions than the experts, mostly advancing the catheter without a guide wire. There was no clear evolution across the attempts. There were on average 2 minor interventions (oral help) per hands-on session for the trainees versus once among the experts. Major interventions (physical help) were rarely needed.

We performed a contrasting group assessment on fluoroscopy and procedure time, the outcome parameters that were statistically significant between trainees and experts, in order to be able to differentiate between both groups. The first attempt was excluded as this was the familiarization session.

**Fig. 3:**
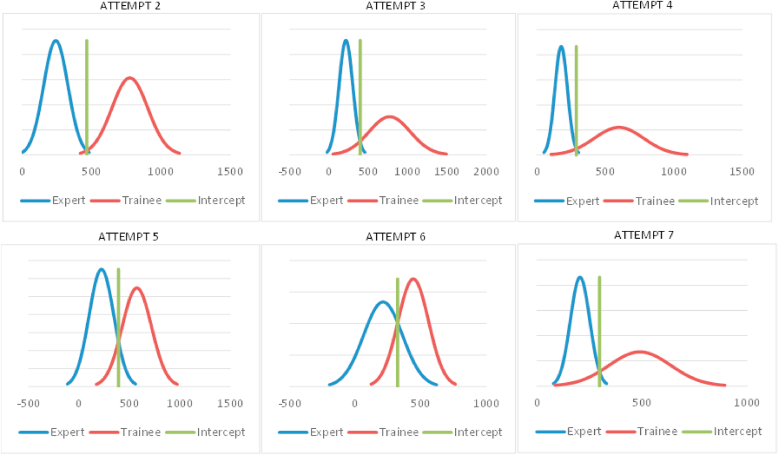
Contrasting group assessment on fluoroscopy time.

Fig. 3 shows the contrasting group assessment for fluoroscopy time for diagnostic angiography. In the second attempt, we observed little overlap between both curves and almost no trainees reached the cutoff. There was a low number of theoretical false negatives (0.9 %). However, the observed false negatives were 5/22 (22.7 %) with the pass/fail cutoff of 465; this discrepancy can be explained by the small sample size (Jørgensen et al., 2018).

As the trainees performed more attempts, the curves of the trainees started to become more widely spread until overlap is observed at the 5th and 6th attempt. The theoretical false negative value at the 6th attempt was at 15.4 % of the trainees. In practice, 9 trainees (40.9 %) reached the level for the pass/fail cutoff.

The curve diverges again at the 7th attempt, showing again a lower theoretical false negative value (9.6 %), as this is the first and only attempt where the trainees no longer have guidance from the local study coordinator and must complete the task entirely autonomously.

**Fig. 4:**
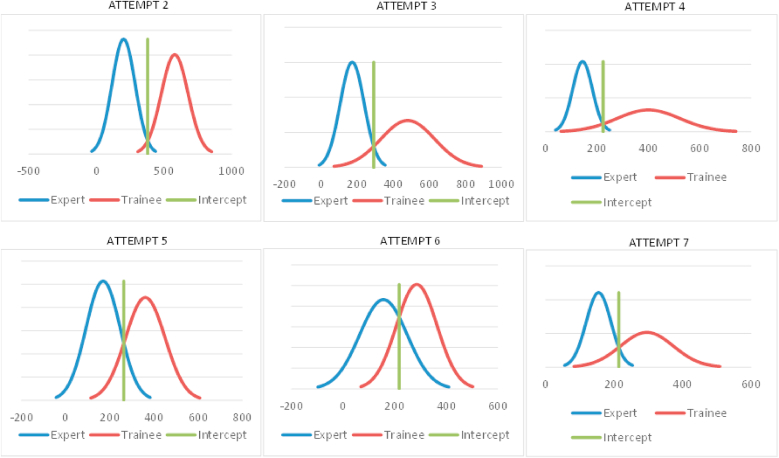
Contrasting group assessment on procedure time.

The similar trend can be seen for procedure time in the diagnostic angiography (see Fig. 4). There were 19.3 % theoretical false negatives at the 6th attempt (pass/fail cutoff of 218) which is consistent with the observed false negatives of 22.7 % (5/22 trainees).

## 4 Discussion

This study evaluated the use of a simulator for neurointerventional procedures. We demonstrated a clear steep learning curve for the trainees regarding procedure and fluoroscopy time for both diagnostic angiography and coiling. This learning curves flattened out as the trainees master both angiography and coiling.

There was a significant improvement of the trainees in both procedure and fluoroscopy time after 7 attempts for diagnostic angiography, with an increasingly narrow discrimination from the experts. The same trend was seen in coiling where there were only 4 attempts. The trend of performance shown in Fig. 1, Fig. 2 are highly illustrative of the effect of simulator-based training.

The contrasting group assessment for fluoroscopy time for diagnostic angiography was also very instructive. The curve first becomes more widely spread, meaning that not every trainee had an equally steep learning curve. But theoretically 15 % of trainees might be able to achieve the pass/fail cutoff at the 6th attempt.

Interestingly, the curves diverge again at the 7th attempt which is at first entirely autonomously. This demonstrates that, after seven attempts, the trainees were capable of performing at a high level—though this was only possible with effective supervision. However, 7 attempts of angiography may not be sufficient to achieve an expert level. Statistically, it is important to note that the contrasting group assessment primarily serves to discriminate between the two groups based on a theoretical cutoff. This cutoff should not automatically be considered the benchmark as this should be determined more by expert opinion (Jørgensen et al., 2018).

The trainees require more practice than the hands-on sessions in the study offered in order to reach a level of proficiency comparable to that of the experts. However, there was no significant difference in safety defined as catheter advancements without wire or contrast use between both groups.

The contrasting group assessment demonstrated discriminating results of experts in comparison with trainees. The outcomes of the experts were consistent across attempts without significant differences and a continuous narrow curve. This indicates that the simulator is a valid tool to demonstrate expertise.

Moreover, the contrasting group assessment showed a distinctly increasing overlap between trainees and experts. Upon determination of the theoretical pass/fail cutoff, there were increasing false negatives regarding procedure and fluoroscopy time. False negatives refer to trainees who still achieve sufficient level to meet the pass/fail cutoff (Jørgensen et al., 2018).

The theoretical false negative value is representative for a large population, for example when implemented in the training program of residents. The discrepancy of the observed and theoretical false negatives in the study can be explained by the small sample size. We believe that the number of trainees achieving the cutoff would increase with the number of attempts, considering the observed trend in the study, although, as stated earlier, this cutoff should not necessarily be the level for benchmarking (Jørgensen et al., 2018).

Therefore, we can conclude that simulator-based training is effective for technical skill evaluation and acquisition for neuroendovascular procedures, being the aim of the study. This research confirms the role of the endovascular simulator potential integration into the angiography training curriculum.

The concept of the learning curve plays a pivotal role in surgical training. While many studies examine learning curves for experienced surgeons, there is less research on trainee learning curves. Competency-based education seems more adequate to assess training then traditional case minimums because they do not take into account individual learning rates. However, assessing a trainee's competence is even more challenging than tracking experience. Reliable data collection is crucial for understanding learning curves, video technology or artificial-intelligence approaches will allow for automated assessment (Toale et al., 2023).

In the context of an endovascular simulation, the learning curve is closely tied to the selected level of procedural difficulty. In our study, a relatively simple scenario is chosen, such as aneurysm coiling without complications. As a result, performance improvements occur early, leading to an early flattening of the learning curve once basic proficiency is achieved. In contrast, if more challenging simulation settings are introduced (for example, complex anatomy, unstable aneurysms, or procedural complications), the cognitive and technical demands may exceed the learner's current skill level, potentially preventing the emergence of a steep learning curve within the same training timeframe. This observation aligns with findings from experimental surgical training literature, such as a recent study, in which trainees performed complex microvascular anastomoses in animal models (Lefevre et al., 2022). In that setting, the high procedural complexity and biological variability resulted in a slower, less predictable improvement pattern, emphasizing that increased task difficulty can obscure or delay measurable learning effects. Together, these findings underscore that learning curves are not solely learner-dependent but are strongly influenced by the inherent complexity of the simulated task.

The use of simulators in training specifically for neuroendovascular procedures represents a transformative development in medical education, addressing many challenges associated with mastering this highly specialized and technically demanding field. One of the most significant advantages of simulator-based training is the ability to create a completely risk-free environment for practice. This allows trainees to acquire and refine their skills but also to make mistakes, an essential part of the educational process, without endangering patient safety. The controlled setting also facilitates unlimited repetition of procedures, enabling learners to achieve confidence and competence over even the complex techniques. As modern simulators are equipped with advanced technology that provides immediate and objective feedback, the trainees can pinpoint specific areas for improvement and track their progress over time. Such feedback also enables instructors to tailor their teaching to address individual trainee needs.

Despite these substantial benefits, simulator-based training might have drawbacks. One limitation is the initial cost of acquiring and maintaining high-fidelity simulators. These costs can be a significant barrier, particularly for smaller or resource-limited institutions. However, simulators may offer long-term cost savings by reducing the costs for training e.g. personnel costs and the use of consumables (direct cost saving) and minimizing the financial risks of training-related errors (indirect cost saving). Additionally, while simulators have made great strides in replicating real-life procedures, they still fall short in certain areas for example the tactile feedback, difficult replication of variabilities in anatomy and absence of the emotional and psychological aspects of neuroendovascular procedures (stress, time pressures, and decision-making challenges). These limitations can lead to a potential gap between skills acquired in simulation and those required in real-world practice.

There are also some limitations to this study in particular. First, the sample size was relatively small, which reduces the statistical power and generalizability of the results. The trainees underwent only a limited number of training sessions within a short timeframe, which restricts our ability to assess the long-term effectiveness of the training. The assessment process is crucial; although most evaluations were standardized through the simulator's automatic controls, and instruction videos for the local coordinators, some assessments were carried out by local coordinators, with potential subjectivity. Lastly, the study did not include self-assessments of trainees' self-confidence, an important factor in skill acquisition that could influence learning outcomes.

Finally, although the results of this study are promising, the validity of simulator assessments in evaluating overall competency and predicting skill transfer to clinical practice remains an area of ongoing research. While simulators provide detailed performance metrics, such as time taken to complete a procedure or the accuracy of tool placement, these measurements may not fully capture a trainee's readiness to perform independently in real-world settings. Further studies are needed to validate the effectiveness of simulators in producing clinically competent neuroendovascular specialists.

## 5 Conclusion

Training on endovascular simulators significantly improves the skills of trainees with notable reduction in procedure and fluoroscopy time, resulting in a steep learning curve and approaching the level of the experts at attempts with accompanying supervision. Endovascular simulators enable skill acquisition in a controlled environment, enhancing technical proficiency and confidence. They should play a role in neuroendovascular training.

## Conflict of interest

Surgical Science provided the simulator for this study and contributes to the EANS Basic Endovascular Course for Neurosurgeons.
