# Supplementary material for: Validation of simulator-based neuroangiographical training
Source: Brain Spine. 2026 Jan 8;6:105931. doi: 10.1016/j.bas.2026.105931 (PMC12828531; doi:10.1016/j.bas.2026.105931)
Supplement: Multimedia component 1 [file mmc1.docx]

# Supplementary material

# 9.1 Supplementary table A: Interventions by study coordinator

|  | **Trainees** | | | |
| --- | --- | --- | --- | --- |
|  | **Diagnostic** | | **Coiling** | |
|  | **Minor** | **Major** | **Minor** | **Major** |
|  | 0 | 0 | 0 | 0 |
|  | 0 | 0 | 1 | 0 |
|  | 0 | 0 | 1 | 0 |
|  | 2 | 0 | 1 | 0 |
|  | 1 | 0 | 1 | 0 |
|  | 0 | 0 | 3 | 0 |
|  | 1 | 0 | 0 | 0 |
|  | 2 | 0 | 1 | 0 |
|  | 2 | 0 | 2 | 0 |
|  | 3 | 0 | 0 | 0 |
|  | 2 | 0 | 2 | 0 |
|  | 2 | 0 | 0 | 0 |
|  | 0 | 0 | 3 | 0 |
|  | 3 | 0 | 1 | 0 |
|  | 1 | 0 | 5 | 0 |
|  | 4 | 0 | 4 | 1 |
|  | 3 | 0 | 1 | 0 |
|  | 3 | 0 | 3 | 2 |
|  | 5 | 0 | 3 | 2 |
|  | 3 | 0 | 3 | 1 |
|  | 2 | 0 | 2 | 1 |
|  | 5 | 1 | 1 | 2 |
| **Total** | **44** | **1** | **38** | **9** |

|  | **Experts** | | | |
| --- | --- | --- | --- | --- |
|  | **Diagnostic** | | **Coiling** | |
|  | **Minor** | **Major** | **Minor** | **Major** |
|  | 0 | 0 | 0 | 0 |
|  | 0 | 0 | 0 | 1 |
|  | 0 | 0 | 0 | 0 |
|  | 1 | 0 | 0 | 0 |
|  | 1 | 0 | 4 | 0 |
|  | 0 | 0 | 1 | 0 |
|  | 1 | 0 | 0 | 0 |
|  | 3 | 0 | 1 | 1 |
|  | 0 | 0 | 1 | 0 |
|  | 4 | 0 | 2 | 2 |
| **Total** | **10** | **0** | **9** | **4** |

| **Mean interventions** | | **Trainee** | **Expert** |
| --- | --- | --- | --- |
| Diagnostic | minor | 2 | 1 |
|  | major | 0 | 0 |
| Coiling | minor | 2 | 1 |
|  | major | 0 | 0 |
| Total | minor | 2 | 1 |
|  | major | 0 | 0 |
